# Identification of tipifarnib sensitivity biomarkers in T-cell acute lymphoblastic leukemia and T-cell lymphoma

**DOI:** 10.1038/s41598-020-63434-5

**Published:** 2020-04-21

**Authors:** Ruth Alonso-Alonso, Rufino Mondéjar, Nerea Martínez, Nuria García-Diaz, Cristina Pérez, David Merino, Marta Rodríguez, Anna Esteve-Codina, Berta Fuste, Marta Gut, Francis Burrows, Catherine Scholz, Jose Pedro Vaqué, Antonio Gualberto, Miguel Ángel Piris

**Affiliations:** 1 Departamento Hematopatología Translacional, IDIVAL, Instituto de Investigación Marqués de Valdecilla, Santander, Spain; 2 Centro de Investigación Biomédica en Red de Cáncer (CIBERONC), Madrid, Spain; 3 Departamento Anatomía Patológica, Fundación Jiménez Díaz, Madrid, Spain; 4 UGC Laboratorios, Hospital Universitario Puerto Real, Cádiz, Spain; 5 Departamento de Biología Molecular, Universidad de Cantabria. Infección, Inmunidad y Patología Digestiva, Instituto de Investigación Marqués de Valdecilla-IDIVAL, Santander, Spain; 6 Unidad Citometría de Flujo, IDIVAL, Instituto de Investigación Marqués de Valdecilla, Santander, Spain; 7 CNAG-CRG, Centre for Genomic Regulation, Barcelona Institute of Science and Technology, 08028 Barcelona, Spain; 8 Centro Nacional de Análisis Genómico (CNAG), Parc Científic de Barcelona, Barcelona, Spain; 9 Kura Oncology, Inc., San Diego, CA USA

**Keywords:** Genetics, Molecular biology, Biomarkers, Molecular medicine, Oncology, Cancer, Cancer genetics, Cancer genomics, Haematological cancer

## Abstract

Patients diagnosed with T-cell leukemias and T-cell lymphomas (TCLs) still have a poor prognosis and an inadequate response to current therapies, highlighting the need for targeted treatments. We have analyzed the potential therapeutic value of the farnesyltransferase inhibitor, tipifarnib, in 25 TCL cell lines through the identification of genomic and/or immunohistochemical markers of tipifarnib sensitivity. More than half of the cell lines (60%) were considered to be sensitive. Tipifarnib reduced cell viability in these T-cell leukemia and TCL cell lines, induced apoptosis and modified the cell cycle. A mutational study showed *TP53*, *NOTCH1* and *DNMT3* to be mutated in 84.6%, 69.2% and 30.0% of sensitive cell lines, and in 62.5%, 0% and 0% of resistant cell lines, respectively. An immunohistochemistry study showed that p-ERK and RelB were associated as potential biomarkers of tipifarnib sensitivity and resistance, respectively. Data from RNA-seq show that tipifarnib at IC_50_ after 72 h downregulated a great variety of pathways, including those controlling cell cycle, metabolism, and ribosomal and mitochondrial activity. This study establishes tipifarnib as a potential therapeutic option in T-cell leukemia and TCL. The mutational state of *NOTCH1*, p-ERK and RelB could serve as potential biomarkers of tipifarnib sensitivity and resistance.

## Introduction

T-cell lymphoproliferative disorders are a very heterogeneous group of lymphoid neoplasms characterized by the clonal expansion of mature T-lymphocytes in bone marrow, blood or other tissues. They include peripheral T- cell lymphoma (PTCL) and T-cell acute lymphoblastic leukemia (T-ALL).

PTCL accounts for about 10% of all non-Hodgkin lymphomas (NHLs)^1^. It comprises a set of disorders that includes peripheral T-cell lymphoma not otherwise specified (PTCL-NOS), angioimmunoblastic T-cell lymphoma (AITL), anaplastic lymphoma kinase (ALK-) positive and ALK-negative forms of anaplastic large cell lymphoma (ALCL), and enteropathy-associated TCL^2^. Despite the subtypes being very different at the molecular level, their treatment is basically similar, consisting of CHOP (cyclophosphamide, doxorubicin, vincristine, and prednisone) and Etopoxide, or a similar regimen. Therapeutic responses to this treatment show a current general 5-year survival rate of only 10–30% and a rapid progression in some subtypes^3–5^.

T-cell acute lymphoblastic leukemia (T-ALL) is an aggressive hematological malignancy characterized by an accumulation of immature T-cell lymphoblasts in bone marrow and peripheral blood. In contrast with PTCL, clinical progress has clearly improved in T-ALL; the 5-year survival rate is currently 65–70%^6^. These results highlight the urgent need for alternative treatment strategies focusing on molecular characterization of individual cases that could identify potential candidates for targeted therapy.

The classification of T-cell lymphomas/leukemias is complex and requires the integration of information about clinical, pathological and genetic factors, morphology, immunohistochemistry (IHC), flow cytometry, cytogenetics, and molecular biology^7,8^. The relatively poor prognosis for PTCL^4^ is ultimately the consequence of our insufficient knowledge about the molecular pathogenesis of these tumors and the limited availability of therapeutic tools^8^.

Deregulated survival pathways and mutated genes have been partially characterized in PTCL. Genetic alterations affecting TCR signaling operate as a common pathogenic mechanism in several TCL entities. Mutations in genes related to TCR co-stimulation and signaling were recently reported in a range of TCL entities, such as cutaneous T-cell lymphoma (CTCL), PTCL-NOS, AITL and ATLL (adult T-cell lymphoma/leukemia). Nonetheless, mutations in specific genes have a variable frequency in distinct entities^9–13^, and, for a given gene, the distributions of the mutations and their relative prevalence are quite variable.

The mutational landscape of T-ALL has recently been elucidated too. A variety of somatic mutations was described like, *JAK1, JAK3, PTEN, IL7R* and *NRAS* signaling proteins^14,15^. Other common oncogenic lesions are the loss of the *CDKN2A* (p16) locus and aberrant *NOTCH1* signaling^16–18^. Activating mutations in *JAK1, JAK3* or *IL7R* lead to activation of the JAK/STAT pathway, resulting in the stimulation of proliferation and survival pathways in the leukemic cells and thereby the development of T-ALL^19,20^.

Aberrant activation of oncogenic Ras signal transduction is a very frequent finding in PTCL and T-ALL^15,21^. Mutations in *RAS* family genes induce constitutive activation of RAS-mitogen-activated protein kinase (MAPK), which activates several downstream effectors that play a role regulating a variety of cell functions, including cell growth, survival and differentiation. In view of these findings and the molecular landscape of PTCL and T-ALL, we were prompted to investigate the Ras mutations and MAPK pathway activation further. Farnesyltransferase inhibitors (FTIs) were designed to disrupt Ras farnesylation and the membrane localization necessary for Ras function. This work and other studies have demonstrated activity in neoplasms lacking mutant Ras^22,23^, suggesting that it could inhibit farnesylation of multiple proteins, leading to the arrest of proliferation and the induction of apoptosis in a variety of preclinical models^24,25^.

Some phase I trials using tipifarnib have demonstrated its antineoplastic effects in solid tumors^26,27^ and leukemia^22^. A variety of phase II trial has shown that it improves early survival when administered as maintenance therapy in patients in remission^28^. Administered as a single-agent, tipifarnib can produce antitumor effect in pretreated patients^29^.

The present study evaluates the effect of inhibiting FTase with tipifarnib^30,31^ in a panel of 25 TCL and T-ALL cell lines, enabling us to determine the therapeutic value of tipifarnib in these cell lines, and to identify biomarkers that could predict the response to this drug and to measure the dynamic effects on cell viability, apoptosis, cell cycle and gene expression. These observations could facilitate the development of personalized therapy in patients with TCL and T-ALL. The selected panel of 25 cell lines includes cell lines derived from a wide array of T-cell lymphoproliferative disorders, including T-cell acute lymphoblastic leukemia (T-ALL), cutaneous T-cell lymphoma (CTCL), anaplastic large cell lymphoma (ALCL) and adult T-cell lymphoma/leukemia (ATLL).

## Results

### T-cell leukemia/lymphoma cell lines are sensitive to tipifarnib

**Figure 1:**
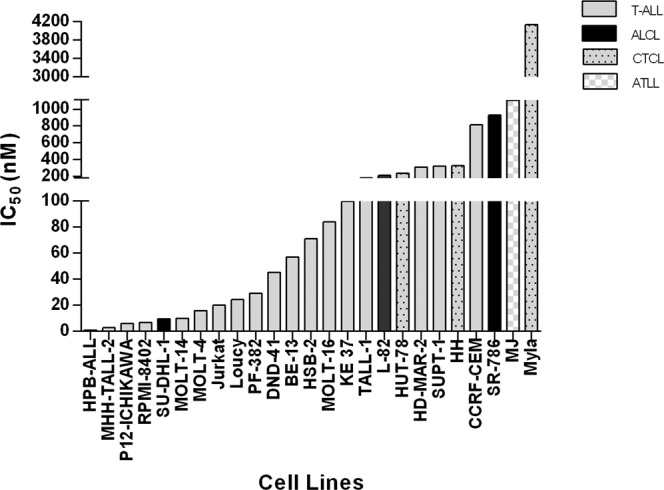
Response of T-cell lymphoma cell lines to tipifarnib after 96 h. IC_50_ values (nM) of the cell lines, in ascending order. Data were treated and the image obtained with Graphpad Prism v5.

Tipifarnib was tested in 25 cell lines (Fig. 1). Tipifarnib readily achieved a peak concentration of 100 nM in the clinic. We categorized cell lines as sensitive (<100 nM) or resistant (≥100 nM) based on IC_50_ values. With this classification, we found 60% of cell lines were sensitive to tipifarnib after 96 h (Fig. 1 and Supplementary Table S1).

### Tipifarnib decreases cell viability, increases apoptosis and blocks cell cycle progression

**Figure 2:**
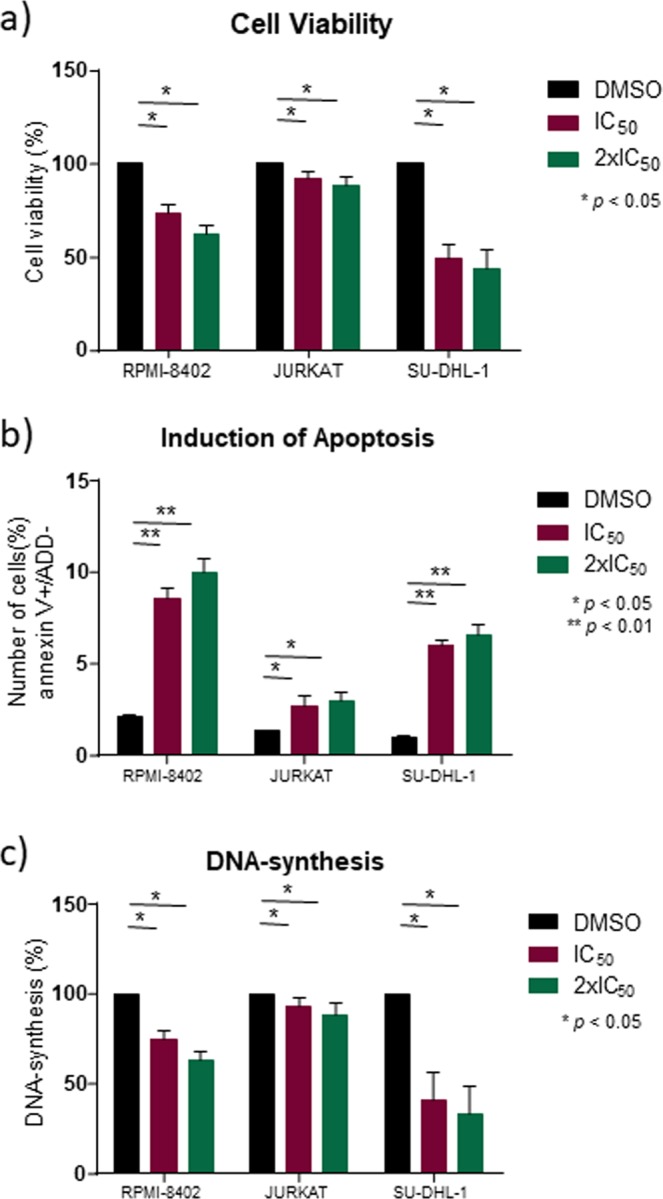
Cell viability and induction of apoptosis in tipifarnib-sensitive cell lines (percentage relative to DMSO). Cell lines were incubated for 96 h at 1x IC_50_ (in purple) and 2x IC_50_ (in green) of tipifarnib, and DMSO (in black) as a vehicle. Each experiment was done in triplicate. Error bars are shown (s.e.m.) (**a**) Tipifarnib reduces cell viability. (**b**) Ability of tipifarnib to increase apoptosis in these lines. (**c**) Percentage of total DNA synthesis in exponentially growing tipifarnib-sensitive cell lines. Probabilities are those associated with Fisher’s exact tests.

Three of the most sensitive cell lines were selected to test cell viability and induction of apoptosis by flow cytometry. These cell lines differ in terms of subtype and mutational level. Jurkat and RPMI-8402 are derived from T-ALL, while SU-DHL-1 is derived from an ALCL. We found that the exposure to tipifarnib after 96 h at the previously calculated IC_50_ value reduced cell viability in these lines (Fig. 2a). We then analyzed the apoptotic effect and found this to be strong in SU-DHL-1 and RPMI-8402 cells (Fig. 2b). We decided to examine whether the drug had any effect on cell cycle progression. We found that tipifarnib was able to inhibit DNA synthesis and thereby block cell-cycle progression in G1 phase, preventing cells from reaching the cellular replication phase (G2 phase) (Fig. 2c). The strongest blockade was observed in the SU-DHL-1 cell line. The JURKAT cell line showed a smaller decrease in cell viability and a lower level of blockade of cell cycle progression, but these were nevertheless significant (p < 0.05).

### Tipifarnib does not change the T-cell phenotype

The T-cell phenotype in PTCL and T-ALL is at least partially regulated by precise mutational events that determine the expression of specific differentiation signatures^32^. Here we have examined whether tipifarnib induces changes in the T-cell phenotype. For this purpose, we studied possible changes in the TH1 (IL2), TH2 (IL-4), Th17 (IL-17) and Treg (Foxp3) phenotypes by analyzing changes in the expression of their markers in eight cell lines: MOLT-14, RPMI-8402, SU-DHL-1, L-82, HPB-ALL, HUT-78, JURKAT and HH. Those cytokines that allow the characterization of inflammatory, non-inflammatory and regulatory subpopulations were measured (Th1, Th2, Th17 and T Reg). No phenotypic changes were observed when cells were cultured in the presence of tipifarnib at IC_50_ and 2x IC_50_ (Supplementary Table S9). From this, we inferred that tipifarnib induces neither an increase in the regulatory phenotypes nor any differentiation into cells with inflammatory characteristics (Th1, Th17). Subsequent studies will determine whether the application of tipifarnib produces other changes in the cell lines, but we have verified that they do not produce modifications that lead to inflammatory or regulatory subtypes.

### Mutational profile and transcriptomic signature are associated with tipifarnib sensitivity

In order to better understand the response to tipifarnib, we performed RNA-seq in a panel of 25 cell lines under basal conditions. In a first analysis, we selected a model that included the IC_50_ value (as a continuous variable), the mutational state of *NOTCH1*, ERK activation (p-ERK; by IHC), sex and cell subtype. In this case, the magnitude of the change represents a coefficient that increases with the IC_50_ value (Supplementary Table S8), which means that a positive value of change is related to resistance, and *vice versa*. For an exploratory view, a false discovery rate (FDR) of <0.25 was used^33^ to generate testable hypotheses. The results suggested that the TCR pathway, G protein-coupled receptor activity and G2/M checkpoints were correlated with highly sensitive cases, while NFkB activation by the TNFα, IFNγ and IFNα response, ribosome and p53 pathway were correlated with resistant cell lines (Supplementary Tables S5–S8).

**Figure 3:**
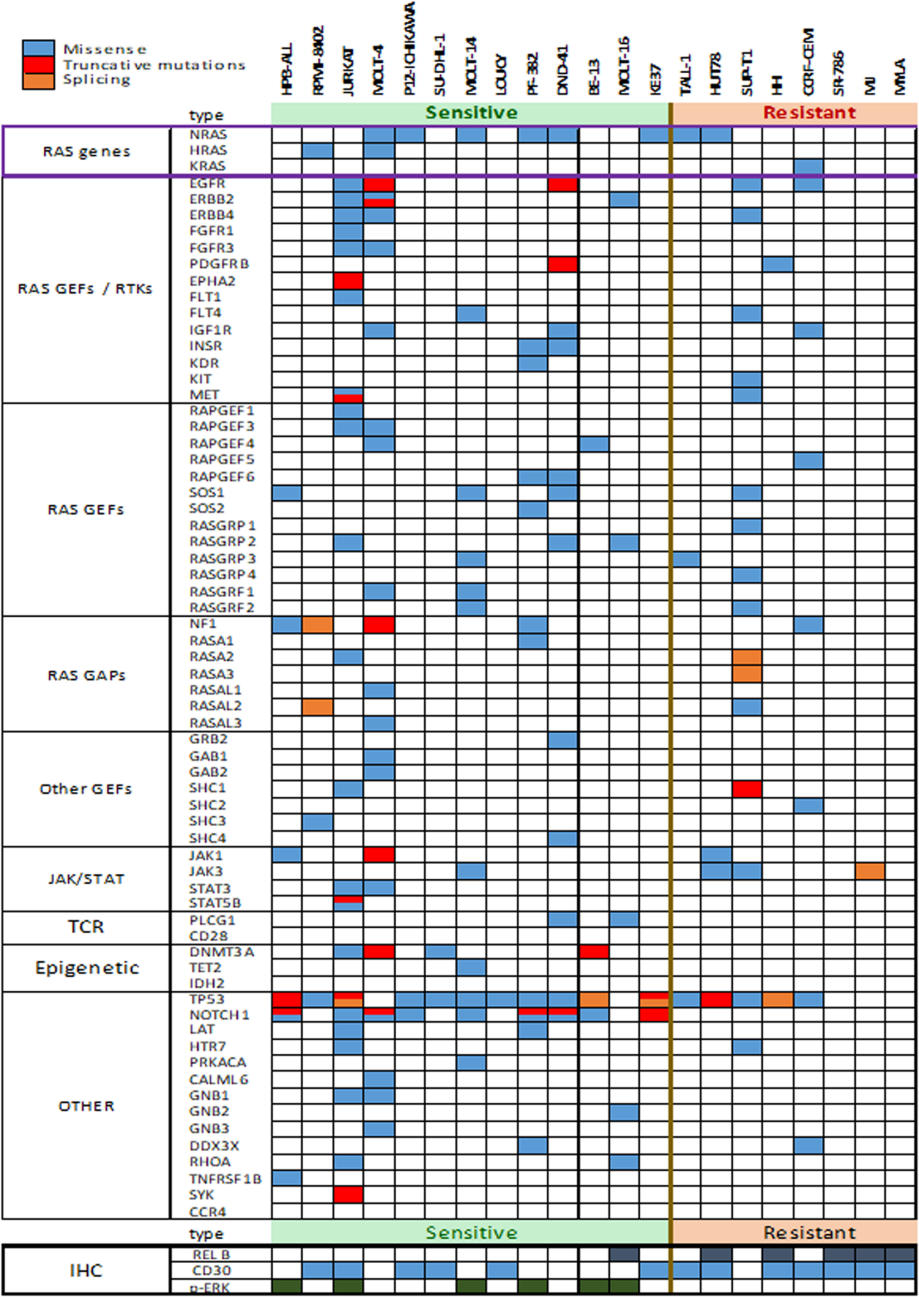
Mutational landscape of RAS/MAPK, JAK/STAT, TCR pathways and T cell lymphoma-related genes in TCL cell lines and IHC markers. Cell lines were grouped into highly sensitive (in green) and less sensitive (in red) in the upper part of the table. Missense mutations are marked in blue, truncated mutations in red, and splicing mutations in orange in the upper part of the table. In the part referring to IHC, dark blue, light blue and green correspond to positive labeling for TP53, CD30 and p-ERK, respectively.

**Figure 4:**
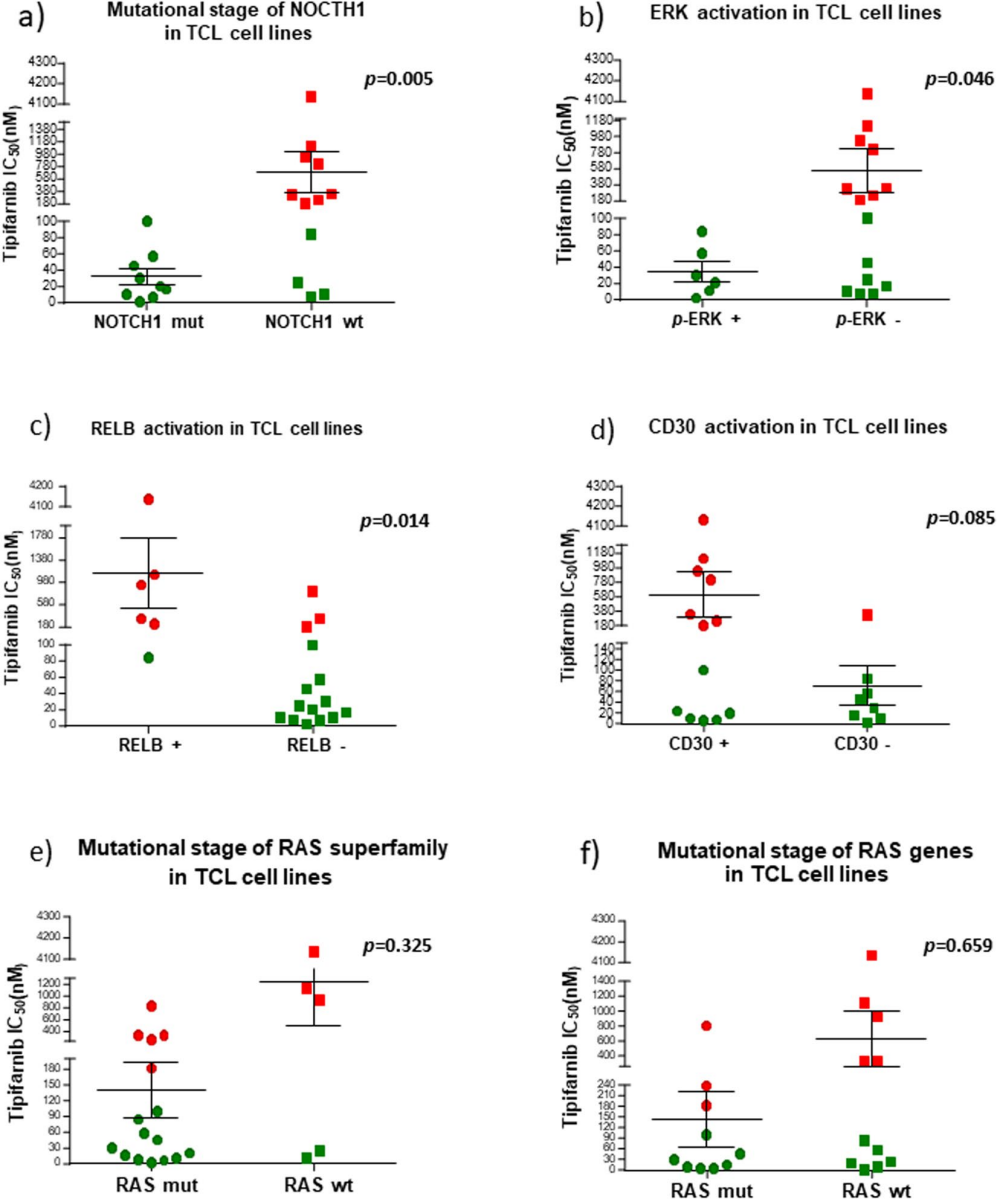
Mutational stage of some biomarkers of tipifarnib sensitivity and resistance in TCL cell lines. (**a**) *NOTCH1;* (**b**) p-ERK*;* (**c**) RELB; (**d**) CD30; (**e**) RAS superfamily; (**f**) RAS genes. Green boxes represent the most tipifarnib-sensitive cell lines; red boxes indicate cell lines with higher IC_50_ (resistant). Graphs were obtained by Graphpad Prism v5 and the probability associated with Fisher’s exact test.

We also analyzed some intracellular signaling pathways associated with T-cell leukemia/lymphoma under basal conditions by IHC. We found an association between ERK activation status and tipifarnib sensitivity (p = 0.046; Figs. 3 and 4b). Conversely, RelB expression was associated with resistance (p = 0.014) (Figs. 3 and 4c).

These results prompted us to focus our analysis on the RAS/MAPK pathway and other T-cell lymphoma pathways, such as TCR, JAK/STAT, NFAT and NFκB. We selected only cell lines with genomic data available from COSMIC or CCLE or generated by our group (Supplementary Table S3). We found that seven (53%) sensitive and three (37.5%) resistant cell lines harbored mutations in *RAS* genes (p > 0.05) (Fig. 4f). 52% and 33% of cells harbored mutations in *RAS*-guanine nucleotide exchange factors (GEFs) and *RAS*-GTPase activating protein (GAP) genes, respectively (p > 0.05, in both cases). The mutational state of the *RAS, RAS-GEF* and *RAS-GAP* genes was not associated with drug sensitivity (Figs. 3 and 4e), which may be explained by the potential effect of tipifarnib on multiple farnesylated genes and pathways. The mutational status of all the *RAS-GEF* genes was associated with ERK activation and negativity of RelB (p = 0.012 and p = 0.040, respectively).

### Tipifarnib downregulates pathways involved in the development of TCL and T-ALL

**Figure 5:**
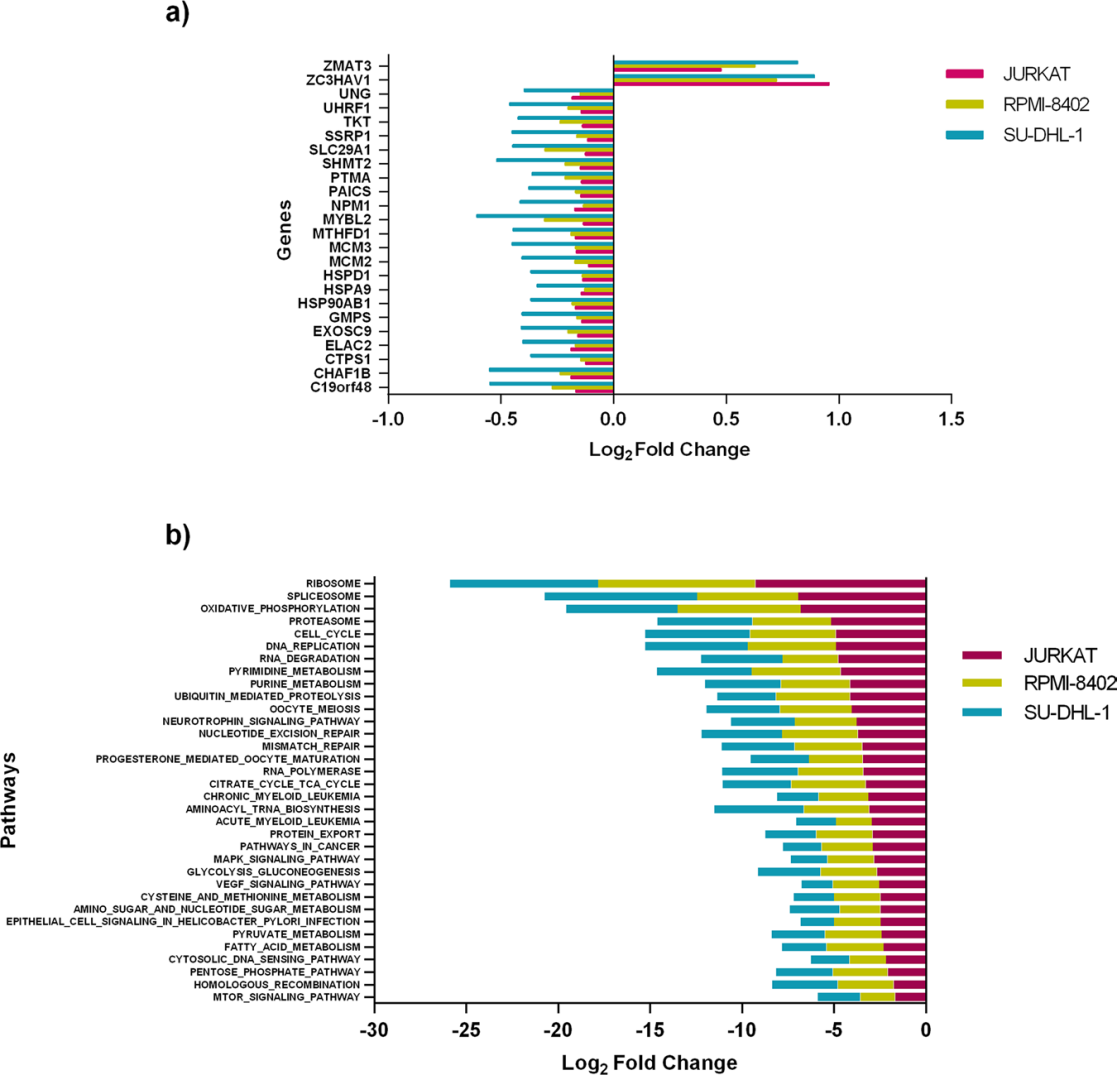
DE genes and pathways commonly regulated by tipifarnib in JURKAT (pink), RPMI-8402 (yellow) and SU-DHL-1 (blue) cell lines. (**a**) 24 genes commonly downregulated (left) and upregulated (right) with tipifarnib, with adjusted values of p < 0.05. Bars show log-fold change. (**b**) Pathways downregulated by tipifarnib. Normalized enrichment score (p < 0.05; FDR < 5%). Bars show log-fold change.

To elucidate the consequences of farnesyltransferase inhibition in TCL and T-ALL cell lines, we performed RNA-seq in three highly sensitive cell lines (JURKAT, RPMI-8402 and SU-DHL-1). Differential gene expression was analyzed with and without tipifarnib. We detected 24 differentially expressed (DE) genes common to all three cell lines (Fig. 5a and Supplementary Table S8).

Two of these upregulated genes are related to damage response and the prevention of infection by retroviruses *(ZMAT3* and *ZC3HAV1*, respectively). Several of the downregulated genes are involved in the cell cycle: *UNG* prevents mutagenesis; *UHRF1* regulates chromatin structure and gene expression; *SSRP1* affects nucleosome disassembly and transcription elongation; *MYBL2* influences cell-cycle progression; *MCM2* and *MCM3* are involved in replication. Other deregulated genes are closely related to enzymatic and mitochondrial activity (*SHMT2, MTHFD1* and *CTPS1*). The heat shock response genes, *HSPD1, HSPA9* and *HSP90AB1*, are other downregulated genes.

GSEA was performed under more stringent conditions (FDR < 0.05; Supplementary Tables S5–8). Tipifarnib downregulated cell cycle, protein localization to cell membrane, metabolism, and ribosomal and mitochondrial activity. Regarding the REACTOME database, tipifarnib downregulated 137 pathways (Supplementary Table S6), and the GO enrichment analysis showed 32 and 975 positive and negative enrichment pathways, respectively (Supplementary Table S7). The most representative downregulated pathways, according to the degree of KEGG enrichment^34,35^, are shown in Fig. 5b. Analyzing pathways involved in T-cell development, we detected that tipifarnib decreased MYC targets and mTORC1 and MAPK signaling.

## Discussion

T-cell leukemia/lymphomas account for between 10 and 15% of all lymphoid tumors and, in most cases, are characterized by an unfavorable prognosis. Despite our growing knowledge about molecular pathogenesis and the diversity of T-cell leukemia/lymphoma cases, the first line of treatment remains similar in all subgroups. The effectiveness of this treatment is quite limited, with only around 25% of patients remaining alive without disease 5 years after the initial diagnosis of TCL or T-ALL^6,36^.

Considerable efforts are being made to identify subclasses of TCL and T-ALL that are susceptible to therapy with specific protocols and to distinguish targetable mutated genes and/or activated pathways. Previous studies by several groups have identified the most commonly deregulated mechanisms driving tumorigenesis in TCL and T-ALL, and provided a useful tool for analyzing the interaction between gene mutations and the activation of key survival pathways^8,19,20,37^.

Given the important role in TCR signaling in neoplastic T-cells, we decide to study the RAS/MAPK pathway and its members to elucidate the genomic basis of TCL and T-ALL. Aberrant activation of the oncogenic RAS signal transduction is commonly observed in these entities. FTIs were initially developed to inhibit RAS activation by blocking farnesylation^38^. Some clinical trials^27,29,39^ showed limited activity, probably due to the ability of RAS to become activated through geranylgeranylation^40^, which is an alternative process to farnesylation.

Here we report the effectiveness of the farnesyltransferase inhibitor tipifarnib in a panel of 25 TCL and T-ALL cell lines, integrating *in vitro* sensitivity data with genomic and immunohistochemical biomarker analysis. Our study suggests that tipifarnib could be a potential therapeutic option in T-cell leukemia/lymphomas, given that the majority of lines were sensitive to tipifarnib (60%) at clinically achievable concentrations (i.e., IC_50_ < 100 nM after 96 h).

Analyzing the differences between sensitive and resistant cell lines we found *TP53, NOTCH1* and *DNMT3A* to be mutated in 84.6%, 69.2% and 30.0% of cells in sensitive cell lines, respectively, in contrast to resistant cell lines, in which the mutational frequencies were much lower (62.5% for *TP53*, and 0% for *NOTCH1* and *DNMT3A*) (Figs. 3 and 4a). The association between the mutational status of *NOTCH1* and tipifarnib sensitivity was statistically significant. This could be an interesting target therapy option in these entities since aberrant *NOTCH1* signaling is the predominant oncogenic lesion involved in the pathogenesis of T-ALL^14,41^, and NOTCH1 interference has been proposed by various researchers^17,41^.

We also compared the activation status of some key intracellular signaling pathways associated with T-cell leukemia/lymphoma. We found an association between ERK activation, measuring p-ERK expression, and tipifarnib sensitivity (Figs. 3 and 4b). Conversely, RelB upregulation, probably reflecting activation of the alternative NFκB pathway, was associated with tipifarnib resistance (Figs. 3 and 4c), suggesting that the mutational status of *NOTCH1* and the activation of ERK could serve as potential biomarkers of sensitivity to tipifarnib, and the positivity of RelB (by IHC) could be a biomarker of resistance to the drug. Other studied markers, such as CD30, showed no such significant associations (Fig. 4d).

Given the association between ERK activation and tipifarnib sensitivity, we focused our attention on the RAS/MAPK pathway and other T-cell leukemia/lymphoma-related pathways, such as TCR, JAK/STAT, NFAT and NFκB, which are known to be involved in T-cell leukemia/lymphoma^8,11,13,42^. Considering RAS members, we found that seven sensitive (46%) and three resistant (37.5%) cell lines harbored mutations in *RAS* genes (not significant (n.s.)); eight sensitive (61%) and three resistant (37.5%) cell lines harbored mutations in *RAS*-guanine nucleotide exchange factors (GEFs) (n.s.) and five sensitive (38%) and two resistant (25%) cell lines harbored mutations in *RAS*-GTPase-activating protein (GAP) genes (n.s.). The mutational state of the *RAS, RAS-GEF* and *RAS-GAP* genes was not associated with tipifarnib sensitivity. The mutational state of all the *RAS-GEF* genes was associated with ERK activation and RelB negativity (p = 0.012 and p = 0.021, respectively).

Under basal conditions, RNAseq analysis of 25 cell lines indicated that the TCR pathway, G protein-coupled receptor activity and G2/M checkpoints were correlated with greater sensitivity to tipifarnib, while NFkB activation by TNFα, IFNγ and IFNα response, ribosome and p53 pathway were correlated with resistance. These results replicate and confirm our previous observations that associated mutational status and pathway activation with tipifarnib sensitivity.

To improve our understanding of the biological mechanism of action of tipifarnib, we performed RNA-seq in three of the most sensitive cell lines (JURKAT, RPMI-8402, and SU-DHL-1). We detected more than 300 differentially expressed genes (Supplementary Tables S5–S7), of which 24 changed their expression consistently in the three cell lines (Supplementary Table S8). GSEA of these data showed that tipifarnib downregulated cell-cycle, protein localization to membranes, metabolism, and ribosomal and mitochondrial activity. These findings concerning cell-cycle downregulation and apoptosis were confirmed by cytometry assays (Fig. 2). In the REACTOME database, tipifarnib downregulated 137 pathways (Supplementary Table S6), and the GO enrichment analysis showed 32 positive and 975 negative enrichment pathways (Supplementary Table S7).

The mammalian target of rapamycin (mTOR) is another commonly activated pathway in TCL and T-ALL^43–45^. As part of the mTORC1 and mTORC2 complexes, mTOR has key roles in several pathways involved in human cancer, which has stimulated interest in mTOR inhibitors. This study has shown that tipifarnib decreases MYC targets and mTORC1 and MAPK signaling, especially in the JURKAT and RPMI-8402 cell lines. A potential role mediating mTORC inhibition as a result of administering FTI has been proposed for RAS homologue enriched in brain (Rheb), which is a farnesylated small GTPase, a key protein in the Rheb-Notch-Rheb loop, that positively regulates mTOR signaling^46–49^.

This study suggests that tipifarnib is a potential therapeutic option in T-cell lymphomas and T-cell acute lymphoblastic leukemia. The results of our study suggest that the mutational state of *NOTCH1* and the activation of ERK could serve as potential biomarkers of tipifarnib sensitivity, and a high level of RelB expression could be a biomarker of resistance to the drug. These results require validation in clinical samples or PDXs.

## Material and Methods

### Cell lines

We included 25 TCL and T-ALL cell lines representing tumor types such as T-cell acute lymphoblastic leukemia/lymphoma (T-ALL, n = 18), anaplastic large cell lymphoma (ALCL, n = 3), cutaneous T-cell lymphoma (CTCL, n = 3), and adult T-cell lymphoblastic leukemia (ATLL, n = 1) (Supplementary Table S2, from^8^). All cell lines were purchased or authenticated before use and were tested for mycoplasma (MycoAlert mycoplasma detection kit; Lonza, Basel, Switzerland).

### Cell culture

CCRF-CEM, HH, HUT-78, LOUCY, MJ, MOLT-4, SU-DHL-1 and SUP-T1 cell lines were purchased from the American Type Culture Collection (ATCC) and cultured with Roswell Park Memorial Institute (RPMI) 1640 Medium (Thermo Fisher Scientific) containing 10% fetal bovine serum (Gibco), except for the MJ cell line, which was cultured with Gibco Iscove’s Modified Dulbecco’s Media (IMDM) medium (Thermo Fisher Scientific) containing 20% fetal bovine serum.

BE-13, DND-41, HD-MAR-2, HPB-ALL, HSB-2, JURKAT, KE-37, L-82, MHH-TALL-2, MOLT-14, MOLT-16, P12-ICHIKAWA, PF-382, RPMI-8402, SR-786 and TALL-1 were purchased from the DSMZ German Collection of Microorganisms and Cell Cultures. Except for HSB-2, they were cultured with Roswell Park Memorial Institute (RPMI) 1640 Medium containing different proportions of fetal bovine serum: 10% for DND-41, HD-MAR-2, HPB-ALL, JURKAT, KE-37, L-82, MOLT-14, MOLT-16, P12-ICHIKAWA, PF-382 and RPMI-8402; 20% for BE-13 and MHH-TALL-2; 15% for SR-786 and TALL-1. HSB-2 was cultured with Gibco Iscove’s Modified Dulbecco’s Media (IMDM) medium containing 10% fetal bovine serum.

MyLa was purchased from the European Collection of Authenticated Cell Cultures (ECACC) and was cultured with Roswell Park Memorial Institute (RPMI) 1640 Medium containing 10% fetal bovine serum.

All cells were cultured in CO_2_ incubators at 37 °C in an atmosphere containing 5% CO_2_.

### Drug sensitivity

All cell lines were tested for tipifarnib sensitivity in two steps, for which purpose they were cultured in appropriate medium in T96 multi-well plates (Falcon 96-well flat-bottom TC-treated Imaging Microplates). Individual optimal cell line seeding densities were determined before deriving growth curves with increasing concentrations of tipifarnib (1, 3, 10, 30, 100 and 300 nM, and 1, 3 and 10 μM) using DMSO as the vehicle for 96 h. Six replicates of all experiments were carried out, and IC_50_ (half-maximal response) values were calculated. Tipifarnib was provided by Kura Oncology (San Diego, CA, USA). Growth curves were derived and IC_50_ values determined using the Cell Titer-Glo Luminescent Cell Viability Assay from Promega (Fitchburg, WI, USA) at 0, 24, 72 and 96 h. IC_50_ values were calculated with Graphpad Prism v5 (La Jolla, CA, USA).

### Apoptosis and cell-cycle analysis

Three of the most sensitive cell lines were subjected to apoptosis and cell-cycle analysis. Flow cytometry assays were used for both analyses, using a Flow Cellect Annexin Red Kit (Millipore, Burlington, MA, USA) and a Click-iT Plus EdU Alexa Fluor 647 Flow Cytometry Assay Kit (Invitrogen, C10634), following the manufacturers’ instructions. Cells were seeded in 12-well plates (VWR International, 10062–894) and incubated for 24 h at 37 °C in a humidified atmosphere of 5% CO_2_. Cells were maintained in standard conditions for 24 h, after which they were exposed to IC_50_ and 2x IC_50_ values of tipifarnib and DMSO as a vehicle for 96 h. The positive and negative controls for the next analysis were included. To identify dead and apoptotic cells, 100 μL of Annexin Red Working Solution were added to each 100 μL of cell suspension and samples were incubated for 15 min at 37 °C. Samples were washed and resuspended in Assay Buffer HSC and then 5 μL of 7-ADD were added. Samples were incubated at room temperature (RT) for 5 minutes in the dark before analysis by Fluorescence-Activated Cell Sorting (FACS). Double-positive cells (Annexin-V and 7-ADD) were considered in the late apoptotic stage or necrotic cells, whereas cells that were positive for Annexin-V but negative for 7-ADD were those in early-stage apoptosis; in this study, we were interested in these early apoptotic cells. To assess the effects on DNA synthesis, cells were grown in each appropriate medium and, after treatment with tipifarnib, incubated for a further 2 h with EdU (5-ethynyl-2′-deoxyuridine), which is a nucleoside analog of thymidine that is incorporated into the DNA during active DNA synthesis (Click-iT Plus EdU Alexa Fluor 647 Flow Cytometry Assay, Invitrogen, C10634). Immediately afterwards, cells were prepared for cytometry following the manufacturer’s recommendations. To calculate the percentage blockade of tipifarnib in the G0 phase and to prevent these cell lines from entering the G2 phase of replication, the percentage inhibition of tipifarnib with respect to DMSO was measured.

### Genomic and IHC data

As previously described by our group^8^, we obtained genomic data from genomic repositories from T-cell lymphoma/leukemia cell lines. Specifically, we combined genomic data from CCLE (Cancer Cell Line Encyclopedia, http://www.broadinstitute.org/ccle, accessed April 30, 2017), COSMIC Cell Lines Project (http://cancer.sanger.ac.uk/cell_lines, accessed April 30, 2017), EGAS00001000268 (European Genome-Phenome Archive, https://www.ebi.ac.uk/ega/), exomes produced previously by our group (Supplementary Table S3), and targeted sequencing of 16 T-cell leukemia/lymphoma-associated genes^8^ (Supplementary Table S4). Variants were called using MiSeq Reporter and RUbioSeq.^50^, employing the default settings, and were visually inspected with the Integrative Genomics Viewer (www.broadinstitute.org/igv/). Variants were annotated using the Variant Effect Predictor (GRCh37, http://grch37.ensembl.org/Tools/VEP). Known SNPs with an allelic frequency greater than 1% in public databases (dbSNP138, 1000 Genomes Project, Exome Sequencing Project, Exome Aggregation Consortium) were filtered out.

### Stranded mRNA library preparation and sequencing

As previously described by Mondejar *et al*.^8^, total RNA was extracted using TRIzol reagent (Invitrogen) according to the manufacturer’s protocol. Total RNA was assayed for quantity and quality using the Qubit RNA BR or HS Assay Kit on the Qubit 2.0 Fluorometer (Life Technologies), and the RNA 6000 Nano Assay on a Bioanalyzer 2100 (Agilent). The RNASeq libraries were prepared using TruSeq Stranded mRNA LT Sample Preparation Kit (Illumina, Inc., Rev. E, October 2013). Briefly, total RNA (500 ng) was enriched for the mRNA fraction and fragmented by divalent metal cations at high temperature (the resulting RNA fragments were 80–250 nt, with a maximum peak of 130 nt). To achieve directionality, the second-strand cDNA synthesis was performed in the presence of dUTP. Mondejar *et al*.^8^ described how the blunt-ended double-stranded cDNA was 3′adenylated and Illumina indexed adapters were ligated. The ligation product was enriched over 15 PCR cycles and the final library was validated on an Agilent 2100 Bioanalyzer with the DNA 7500 assay (Agilent). Libraries were sequenced on HiSeq2000 (Illumina, Inc.) in paired-end mode with a read length of 2 × 76 bp using a TruSeq SBS Kit v4 in a fraction of a sequencing v4 flow cell lane, following the manufacturer’s protocol. Image analysis, base calling and quality scoring of the run were processed using the manufacturer’s Real Time Analysis (RTA 1.18.66.3) software, followed by generation of FASTQ sequence files.

### RNA-seq data processing and analysis

RNA-seq was performed in 25 cell lines under basal culture conditions. RNA-seq paired-end reads were mapped against the human reference genome (GRCh38) using STAR version 2.5.3a^51^ with ENCODE parameters for long RNA. Annotated genes (GENCODE version 27) were quantified using RSEM version 1.3.0^52^ with default parameters. Differential expression was analyzed with limma-voom^53^ adjusted for sex, cell subtype and 18 other hidden factors detected by surrogate variable analysis (sva). The IC_50_ was considered as a continuous variable, and only genes with at least 1 cpm in 10 samples or more were selected for the analysis.

To further elucidate the consequences of farnesyltransferase inhibition in TCL and T-ALL cell lines, RNA-seq was performed in three cell lines (RPMI-8402, SU-DHL-1 and JURKAT) treated with tipifarnib for 72 h at IC_50_ and DMSO in three biological replicates. RNA-seq reads were mapped and genes were annotated as described above. Principal component analysis of the ‘rlog’-transformed expression data was done using the top 500 most variable genes with the ‘prcomp’ R function and ‘ggplot2’ R library. Two outlier samples belonging to DMSO_SUDHL1 and TIPI_SUDHL1 were removed. Differential expression was analyzed with DESeq2 version 1.10^54^ for each cell line separately. Heatmaps with the top 50 differentially expressed genes were generated with the ‘pheatmap’ R package. Gene set enrichment analysis (GSEA) was performed from a background of > 5000 gene sets of the Hallmark collection of the Molecular Signatures Database (MSigDB), curated (C2 collection: KEGG, BIOCARTA, REACTOME) and Gene Ontology (GO) gene sets (C5 collection). Expression data have been deposited in the Sequence Read Archive (SRA) under BioProject accession reference PRJNA603759.

### Phenotypic characterization

The expression of the markers IL-2, IL-4, IFN-g, IL-17, RORγT and Foxp3 was tested by FACS in eight cell lines (MOLT-14, RPMI-8402, SU-DHL-1, L-82, HPB-ALL, HUT-78, JURKAT and HH), enabling possible changes in the TH1/TH2/Treg/Th17 phenotypes to be studied. Cell lines were incubated for 96 h at 1x and 2x the IC_50_ of tipifarnib and DMSO in plates of T12 wells (VWR International, 10062-894). All experiments were done in triplicate.

### Statistical analysis

The chi-square or Fisher exact test were used to determine the significance of associations between the presence or absence of markers and sensitivity. Estimates were considered statistically significant for two-tailed values of p < 0.05. All analyses were carried out using IBM SPSS for Windows version 20 (IBM Corp., Armonk, NY, USA).

## Supplementary information

Supplementary Table Legends.

Supplementary Tables.

## Data Availability

The datasets generated during the current study are available in the Sequence Read Archive (SRA) under BioProject accession reference PRJNA603759. We consulted and obtained genomic data from genomic repositories from T-cell lymphoma/leukemia cell lines (see *Genomic and IHC data* in Materials and Methods section).
