# Supplementary material for: Identification of tipifarnib sensitivity biomarkers in T-cell acute lymphoblastic leukemia and T-cell lymphoma
Source: Sci Rep. 2020 Apr 21;10:6721. doi: 10.1038/s41598-020-63434-5 (PMC7174413; doi:10.1038/s41598-020-63434-5)
Supplement: Supplementary file 1 — Supplementary Table Legends. [file 41598_2020_63434_MOESM1_ESM.pdf]

Identification of tipifarnib sensitivity biomarkers in T-cell acute lymphoblastic leukemia and T-cell lymphoma

Ruth Alonso-Alonso[1,2,3, #], Rufino Mondéjar[1,2,4, #], Nerea Martínez[1,2], Nuria García-Diaz[5], Cristina Pérez[1,2], David Merino[6], Marta Rodríguez[2,3], Anna Esteve-Codina[8], Berta Fuste[7], Marta Gut[7], Francis Burrows[9], Catherine Scholz[9], Jose Pedro Vaqué[5], Antonio Gualberto[9] and Miguel Ángel Piris[2,3,*]

SUPPLEMENTARY MATERIAL

Supplementary table S1. Response of T-cell lymphoma/leukemia cell lines to Tipifarnib at 96h.

Supplementary table S2. Cell lines used in the study.

Supplementary table S3. Public data from genomic repositories and our exomes in cell lymphoma/leukemia cell lines.

Supplementary table S4. List of genes sequenced by amplicon-based methodology in cell lines.

Supplementary table S5. KEGG enrichment analysis.

Supplementary table S6. REACTOME enrichment analysis.

Supplementary table S7. GO enrichment analysis.

Supplementary table S8. Differential gene expression of resistant and sensitive cases.

Supplementary table S9. Expression of several phenotypic markers in eight cell lines after exposure to DMSO, 1X IC_{50} and 2XIC_{50} doses of tipifaranib at 96 h. The values correspond to the percentage of positive cells for each marker in relation to the number of total cells in each condition. Control shows values under basal culture conditions.
